# Can gamification really enhance learning performance? The importance of balancing gamification design and content quality

**DOI:** 10.3389/fsoc.2026.1740629

**Published:** 2026-06-19

**Authors:** Zhanhe Du, Tiantian Li, Mengqian Xu, Chenhui Xiong

**Affiliations:** School of Economics and Management, Xi’an University of Technology, Xi'an, China

**Keywords:** content quality, external supervision, gamification design, learning performance, online learning platforms, social learning theory

## Abstract

**Introduction:**

Online learning platforms often integrate gamification and content strategies to improve user engagement and learning outcomes. However, excessive reliance on gamification may hinder performance, especially when content quality is overlooked. This study investigates the joint effects of content quality and gamification design on learning performance and the moderating role of external supervision.

**Methods:**

Drawing on Social Learning Theory, a research model was developed and tested using survey data from 207 users of a leading Chinese online learning platform.

**Results:**

Results show that content quality significantly enhances learning performance, while empirical data suggests a potential inverted U-shaped effect for gamification—beneficial at moderate levels but exhibiting diminishing returns when overused. Moreover, external supervision enhances the positive effects of moderate gamification but exacerbates the negative impact of excessive gamification.

**Discussion:**

The study extends Social Learning Theory by incorporating institutional supervision and provides actionable insights for designers and educators to balance content and engagement strategies in digital learning systems.

## 1 Introduction

Online learning platforms are increasingly navigating a fundamental tension. In an era where digital education has become central to knowledge acquisition, these platforms must balance two core priorities. On one side is content quality—the bedrock of learning that ensures materials are accurate, meaningful, and relevant (Huang et al., 2021; Singh and Thurman, 2019). High-quality content builds trust and ensures that learning is substantive (Kam and Umar, 2023), fostering deep understanding rather than superficial engagement. On the other hand, there is the powerful appeal of gamification, which integrates game-like features such as badges, points, and leaderboards to make the learning process more motivating and enjoyable (Oliveira et al., 2023; Li et al., 2024a). While many platforms invest heavily in both, the balance often shifts toward gamification to capture user attention (An and Wulf, 2024; Roslan et al., 2023). This growing reliance on engagement mechanics, however, raises a critical question: as platforms become more engaging, are they becoming less effective at their primary mission of education?

However, the current understanding of this dynamic in the literature is not yet complete. Previous research often adopts a siloed approach, examining content and engagement features separately (Al-Fraihat et al., 2020; Wang et al., 2024). This is problematic because it overlooks the reality that learners experience these elements simultaneously, leading to potential interactive effects that can either enhance or interfere with their individual benefits. While the link between content quality and learner satisfaction is well-established, its direct impact on measurable learning outcomes within gamified settings remains a subject for deeper exploration. At the same time, research on gamification has yielded inconsistent results (Groening and Binnewies, 2019). Some studies praise its ability to boost motivation (Leung et al., 2023), while others caution that poorly implemented gamification can create cognitive overload (Kwon and Özpolat, 2021), distract from learning objectives, and result in a short-lived engagement that quickly fades as the initial excitement wears off (Tsay et al., 2020). This points to a more complex, nonlinear relationship where more is not always better. Furthermore, a key perspective is frequently overlooked in the existing literature: the active regulatory role of external supervision (Zhang et al., 2024; Dabbagh and Kitsantas, 2012). Social Learning Theory (SLT) posits that learning is fundamentally shaped by triadic reciprocal interactions among environment, cognition, and behavior (Bandura, 1989; Bandura, 1991; Zuchowski, 2015). While traditional applications of SLT emphasize learning as a micro-level social process guided by interactions with teachers and peers, they often fail to account for the broader environmental dynamics of institutional oversight. By overlooking this crucial environmental factor, current research misses how external supervision actively intervenes to recalibrate the self-regulation of learners, particularly when digital engagement mechanics become distracting.

To investigate these issues more deeply, this study develops and empirically tests a holistic model grounded in Social Learning Theory. Using questionnaire data collected from users of a widely used online learning platform in China, we analyze how content quality, gamification, and external supervision interact to shape learning performance. Our findings first confirm that content quality remains the most powerful and reliable predictor of learning success, reinforcing its non-negotiable role as the foundation of any effective platform (Zhang and Huang, 2024; Ho et al., 2023). Second, we provide preliminary empirical evidence that gamification’s impact is not linear but follows an inverted U-shaped curve. This finding helps clarify a persistent debate in the literature by demonstrating that while moderate gamification is beneficial (Cai et al., 2025), excessive use becomes detrimental to learning (Kock and Lynn, 2012; Kwon and Özpolat, 2021). Third, our analysis uncovers the complex moderating role of external supervision. It not only enhances the positive effects of moderate gamification but can also, counterintuitively, worsen the negative impact of over-gamification, highlighting the need for a delicate balance. Practically, these findings offer critical insights for platform designers and educators on how to effectively balance content, gamification, and supervision.

This study advances the theoretical understanding of online learning by extending Social Learning Theory to incorporate institutional supervision as a contextual moderator. While prior studies have largely treated learning as a function of individual motivation and platform design, our findings reveal that supervisory mechanisms play a significant role in shaping how learners respond to gamified features. Furthermore, by identifying an inverted U-shaped relationship between gamification and learning performance, this study provides empirical support for the ‘too much of a good thing’ perspective, demonstrating that engagement-enhancing mechanisms can backfire when overused. Finally, it reinforces the centrality of content quality, highlighting that meaningful learning outcomes ultimately depend on the substantive and credible quality of instructional materials.

The results offer actionable insights for online education practitioners and platform designers. First, developers should prioritize content quality as the foundation for user trust and sustained engagement. High-quality, credible materials encourage learners to invest cognitive effort, leading to better performance. Second, gamification should be implemented with moderation—avoiding excessive competitive or reward-based elements that may shift attention away from learning objectives. Platform designers should emphasize intrinsic rather than extrinsic motivators to maintain engagement without distraction. Third, institutions and educational authorities should adopt supervision mechanisms that balance encouragement and oversight. Effective supervision can guide learners toward optimal engagement levels, ensuring that gamification complements rather than dominates the learning experience.

## 2 Theory and hypothesis

### 2.1 Gamification

Gamification, defined as the application of game elements in non-game contexts, aims to enhance engagement and motivation in fields such as education (Hamari et al., 2015; Aparicio et al., 2019). It leverages game mechanics like challenges, leaderboards, and badges to create enjoyable learning experiences, promoting user engagement and improving learning outcomes (Ding, 2019; Groening and Binnewies, 2019; Blair et al., 2016; Li et al., 2024b). By fostering intrinsic motivation, gamification encourages learners to participate actively and consistently, leading to enhanced critical thinking, satisfaction, and retention (Martínez-Jiménez and Ruiz-Jiménez, 2020).

Despite its potential, the effectiveness of gamification in education is subject to limitations and challenges. Overemphasis on game elements can detract from content comprehension, causing learners to prioritize rewards over substantive learning (Tseng et al., 2024). Furthermore, user responses to gamification features vary widely, influenced by intrinsic motivation and individual learning goals. For instance, while some learners may find gamification engaging, others may perceive it as unnecessary or distracting, reducing its overall impact. This variability highlights the need for a tailored approach to gamification that considers diverse user characteristics and learning objectives. Moreover, gamification alone is insufficient to guarantee effective learning outcomes (Dermentzi, 2024). While it serves as a tool to attract and retain users, its success depends on integration with high-quality content that meets learners’ educational needs (Alyoussef, 2023; Hassan et al., 2021). Platforms with poorly designed gamification or substandard content risk disengaging users and failing to deliver meaningful learning experiences. Studies emphasize that balancing gamification with the intellectual rigor of content is critical for achieving holistic success in online education (Shortt et al., 2021; Deterding et al., 2011).

In conclusion, gamification is a double-edged sword in online learning platforms. When designed appropriately, it can significantly enhance engagement, motivation, and learning efficiency (Özdemir, 2025). However, excessive or poorly implemented gamification may hinder learning outcomes by shifting focus away from educational goals. To maximize its benefits, gamification must be context-specific, aligned with user needs, and seamlessly integrated with high-quality content, ensuring a balanced approach that prioritizes both engagement and educational value (Grech et al., 2024; Martínez-Jiménez and Ruiz-Jiménez, 2020).

### 2.2 Social learning theory

Social Learning Theory (SLT) provides a robust framework for understanding how individuals acquire new behaviors through observing the actions and attitudes of others, as well as the consequences of those actions (Bandura, 1989). This theory has been extensively applied across disciplines, including education and information management, to explain how environmental and cognitive factors interact to shape learning outcomes (Khechine et al., 2020). However, beyond mere observation, the true explanatory power of this theory lies in the concept of triadic reciprocal determinism. This concept highlights a dynamic and continuous interplay among cognitive, behavioral, and environmental factors (Bandura, 1991). Within the context of online learning platforms, the present study leverages this triadic framework to move beyond a descriptive understanding of platform features. Instead, it theorizes exactly how platform design and institutional oversight dynamically recalibrate user cognition and learning behaviors.

Rather than treating platform features as isolated technical components, this study operationalizes content quality and gamification design as deeply intertwined environmental stimuli that drive the mechanisms of SLT. First, high-quality content serves as the foundational cognitive input for observational learning. By providing authoritative knowledge resources, structured materials, and expert problem-solving approaches, high-quality content allows learners to internalize effective learning strategies and construct robust cognitive schemas (Zhang and Shakibaei, 2025). Concurrently, gamification design functions as an environmental reinforcement mechanism. Gamified elements such as points, badges, and leaderboards publicly manifest the achievements of learners and trigger vicarious reinforcement (Coelho et al., 2025). This high visibility motivates users to emulate successful behaviors in order to obtain similar rewards (Shao et al., 2025). Together, the knowledge value derived from content quality and the behavioral incentives provided by gamification form the dual environmental forces that shape learner engagement.

Crucially, while existing studies often describe environmental factors in a passive manner, this research advances SLT by conceptualizing external supervision as an active institutional calibration mechanism within the triadic reciprocal determinism model (Lin et al., 2025). According to the theory, successful learning ultimately relies on the capacity of a learner for self-regulation (Bandura, 1989). However, in highly gamified digital environments, extrinsic stimuli can sometimes overwhelm intrinsic cognitive goals and disrupt this vital self-regulatory process. In this complex dynamic, external supervision acts as an essential boundary-setter (Liao, 2026). Through continuous monitoring, explicit feedback, and structured guidance, external supervision forcefully recalibrates the self-regulation of learners. This intervention ensures that the behavioral drive elicited by gamification remains firmly anchored to the cognitive objectives dictated by the content quality (Zhang and Shakibaei, 2025).

By integrating these theoretical processes, this study overcomes the limitations of descriptive framework applications. It demonstrates how content quality and gamification design actively drive learning performance, while theorizing external supervision as the critical institutional scaffolding necessary to maintain the delicate balance of the triadic reciprocal determinism in digital learning ecosystems.

### 2.3 Content quality and learning performance

Content quality represents the cornerstone of online learning platforms, encompassing learning resources defined by credible information sources, frequent updates, and substantive knowledge. It aligns with the broader concept of information quality, emphasizing reliable knowledge dissemination, diverse material offerings, and regular content updates (Cidral et al., 2018). High-quality content is critical for shaping users’ learning goals and evaluating platforms’ effectiveness in facilitating meaningful learning experiences. Moreover, it plays a pivotal role in user adoption and continued engagement with e-learning platforms (Yang et al., 2017). While prior studies have often assessed course quality or information quality as standalone constructs, these approaches may overlook the broader spectrum of learning resources available on digital platforms. This study extends the concept of content quality to include formal and informal learning resources, as well as interactive content, reflecting the multifaceted nature of modern online education.

To explore the impact of content quality on user learning performance, we identify three key dimensions: authority, timeliness, and knowledge. Authority ensures information reliability by emphasizing expert or certified content, guaranteeing accuracy and professionalism. Timeliness highlights the importance of providing current and regularly updated content, addressing users’ demand for relevant and up-to-date knowledge. Knowledge focuses on the depth and breadth of the information, enabling learners to acquire systematic understanding and apply it effectively. Collectively, these dimensions shape the perceived quality of content, directly influencing user trust, satisfaction, and learning outcomes.

Grounded in SLT, content quality can be understood through observational learning and triadic reciprocal determinism. Observational learning explains how users refine their strategies by observing modeled applications of high-quality knowledge. For instance, content that demonstrates expert problem-solving or provides actionable insights motivates learners to emulate these approaches, fostering deeper engagement. Triadic reciprocal determinism describes the dynamic interplay between content quality, user cognition, and behavior. High-quality content not only provides structured and comprehensive resources that support independent learning but also facilitates active participation, reinforcing key concepts and building learners’ self-efficacy. This increased self-efficacy enhances users’ confidence in their ability to achieve learning goals, leading to improved learning outcomes. Empirical evidence supports the link between content quality and user satisfaction (Lu et al., 2023) as well as its positive influence on learning performance (Zhong and Chen, 2023). High-quality content not only attracts and retains users but also fosters a sense of trust in the platform, driving sustained engagement and meaningful learning achievements. Accordingly, this study hypothesizes:

*H1*: Content quality enhances users' learning performance on online learning platforms.

### 2.4 Gamification design and learning performance

Gamification design in online learning platforms encompasses the inclusion, presentation, and integration of gamification elements with platform content. While the ultimate goal of users engaging with such platforms is to enhance learning performance, gamification design serves as a key facilitator in achieving this objective (Mellado and Cubillos, 2024). Previous research has shown that well-designed gamification strategies can significantly improve the learning experience by fostering user engagement, increasing motivation, and encouraging continuous platform usage. These outcomes collectively contribute to enhanced learning performance (Akrong et al., 2022; Alshammari, 2020). Diverse and rich gamification elements—such as story-based scenarios, leveled Q&A, and interactive dialogues—capture users’ attention, create enjoyable learning experiences, and foster social interactions among users with similar interests (Liu et al., 2024). By integrating game design into the learning process, platforms can alleviate initial feelings of boredom, promote deeper engagement, and motivate learners to achieve their educational goals, thereby improving overall learning outcomes.

However, the relationship between gamification design and learning performance is not linear. While moderate levels of gamification can enhance user experience and learning outcomes, excessive gamification may lead to unintended negative consequences. Studies have demonstrated that as gamification intensity increases, users may shift their focus from learning objectives to gaming incentives, such as earning badges or achieving high leaderboard rankings (Baydas and Cicek, 2019; Tseng et al., 2024). This shift in priorities can detract from knowledge acquisition and reduce the effectiveness of the learning process. Feng et al. (2024) further highlighted that overemphasis on gamification elements can diminish users’ learning performance rather than enhance it. Moreover, as gamification design becomes more elaborate and complex, users may experience a loss of novelty and engagement, resulting in frustration or distraction from the learning process. Excessive reliance on game mechanics can, in such cases, undermine the platform’s educational purpose, creating a paradox where gamification hinders rather than supports learning outcomes.

According to Cognitive Load Theory, individuals possess limited cognitive processing capacity when engaging in learning tasks (Sweller, 2011). Moderate levels of gamification design can activate learners’ attention through engaging elements, thereby directing limited cognitive resources toward core learning content and reducing extraneous cognitive load, which ultimately enhances learning performance (Skulmowski and Xu, 2022). However, excessive gamification may introduce numerous task-irrelevant elements, such as complex point systems and frequent leaderboard notifications (Li et al., 2025). These elements can occupy learners’ attentional and cognitive resources, thereby increasing extraneous cognitive load and interfering with effective information processing (Leppink and van den Heuvel, 2015). As a result, learners may shift their attention away from knowledge acquisition toward reward-oriented activities, which can ultimately undermine learning performance (Baah et al., 2024).

Given these considerations, the relationship between gamification design and learning performance can be conceptualized as an inverted U-shaped curve. Moderate gamification design provides an optimal balance, enhancing engagement and motivation while preserving focus on learning objectives. Beyond this optimal level, however, excessive gamification introduces diminishing returns and may ultimately harm learning performance. Based on the above analysis, we propose the following hypothesis:

*H2*: Platform gamification design has an inverted U-shaped relationship with user learning performance in online learning platforms.

### 2.5 Moderating role of external supervision

External supervision is recognized as a crucial mechanism for quality assurance in the learning process (Kilminster and Jolly, 2000). It involves monitoring and guiding learners through the support of institutions such as schools, departments, or social organizations (An and Wulf, 2024; Roslan et al., 2023). This process not only ensures that learners complete tasks according to established standards but also promotes the achievement of desired learning outcomes and the development of effective learning habits (Wanzare, 2012). In online learning environments, relying solely on high-quality content or gamified design may not fully optimize learning outcomes, as individual differences and environmental influences also play significant roles (Zuchowski, 2015).

Social Learning Theory (SLT) asserts that learning behaviors are shaped by dynamic interactions among environmental factors, cognitive processes, and behavioral responses (Moon and Kim, 2001). In digital learning environments, external supervision—such as instructor monitoring and feedback—serves not merely as a contextual backdrop, but as an active regulatory mechanism (Wong et al., 2019). From the SLT perspective, when excessive gamification lures learners to shift their focus toward reward accumulation, a behavioral deviation, external supervision intervenes to provide corrective feedback (Krath et al., 2021). This intervention strengthens the learner’s self-regulation, a cognitive recalibration, ensuring that limited cognitive resources remain focused on core educational objectives. Consequently, supervision functions as an institutional scaffold that supports the self-regulatory processes central to SLT.

Through the principle of observational learning, external supervision enriches learners’ understanding of complex concepts by providing demonstrations and explanations from instructors or mentors (Bandura, 1991). For instance, when supervisors deconstruct intricate problems and illustrate solution methods, learners not only gain knowledge but also develop problem-solving approaches applicable to similar challenges. Immediate feedback from supervisors addresses misconceptions, ensuring learners remain aligned with learning objectives (An and Wulf, 2024; Roslan et al., 2023). Additionally, SLT’s self-observation process emphasizes how external supervision enhances learners’ self-efficacy. By setting clear goals, offering encouragement, and recognizing achievements, supervisors help learners build confidence and intrinsic motivation (Abbey and Meloy, 2017). As learners accomplish incremental milestones under guidance, their sense of capability grows, fostering sustained engagement and a proactive attitude toward exploring additional resources and challenges. Furthermore, supervision provides constructive feedback tailored to observed behaviors, enabling learners to refine strategies and avoid repeated errors, thereby optimizing learning outcomes (Cools et al., 2013). Grounded in these mechanisms, this study hypothesizes:

*H3*: External supervision positively moderates the relationship between content quality and learning performance.

External supervision plays an important role in shaping the relationship between gamification design and learning performance. Gamification elements such as rewards, points, and leaderboards can stimulate learner motivation and increase engagement with online learning platforms (Aparicio et al., 2019). However, prior studies have also noted that excessive reliance on gamification may shift learners’ attention from meaningful knowledge acquisition toward reward-oriented behaviors, ultimately reducing learning effectiveness (Baydas and Cicek, 2019; Tseng et al., 2024). This suggests that the effectiveness of gamification is contingent not only on its intensity but also on contextual factors that guide how learners interact with gamified elements.

Social Learning Theory (SLT) provides a useful perspective for understanding how external supervision may regulate this relationship. According to SLT, learning behaviors are shaped through interactions among environmental factors, cognitive processes, and behavioral responses. External supervision—such as instructor guidance, monitoring, and feedback—serves as an important environmental cue that can influence learners’ cognition and behavior in digital learning environments. Through observational learning, learners can adopt effective learning strategies demonstrated by instructors or peers, while supervisory feedback helps them adjust their learning behaviors and remain aligned with learning objectives (Huang et al., 2024). In this way, supervision can enhance learners’ self-regulation and encourage goal-oriented engagement with platform features.

Importantly, the role of external supervision may differ across stages of gamification intensity. When the level of gamification is relatively low or moderate, supervision can reinforce learning goals and provide guidance on how to effectively engage with gamified features. Such support may help learners focus their attention on meaningful learning tasks, thereby strengthening the positive effects of gamification on learning performance (Li et al., 2024a). In contrast, when gamification becomes excessive and learners are more likely to focus on reward accumulation or competitive rankings, supervision can serve a corrective function by redirecting learners’ attention toward substantive knowledge acquisition. Through guidance and feedback, supervisors can help learners maintain a balance between gamified incentives and learning objectives, thereby mitigating the potential negative consequences of excessive gamification (Liu and Zhou, 2025).

Based on this reasoning, external supervision is expected to regulate the inverted U-shaped relationship between gamification design and learning performance. This study hypothesizes:

*H4*: External supervision enhances the inverted U-shaped relationship between gamification design and learning performance, leading to higher learning outcomes.

**Figure 1:**
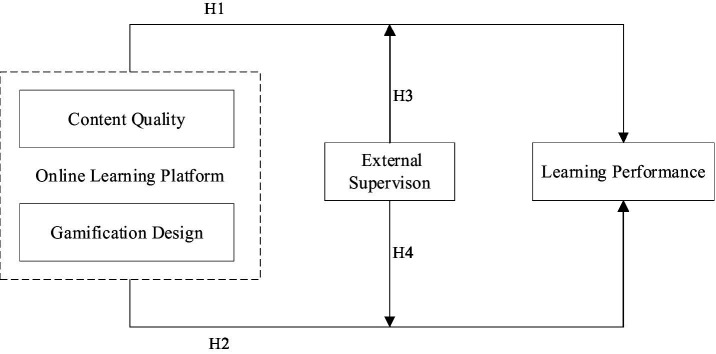
Research model.

The research model that we empirically test is summarized below (Figure 1 Research Model).

## 3 Materials and methods

### 3.1 Data collection

The data for this study were collected from users of Xuexi Qiangguo (LPP, Learning Power Platform), a widely used online learning platform in China. Launched by the Publicity Department of the Communist Party of China, the platform aims to promote lifelong learning through the integration of educational content and digital technology. The platform provides diverse learning materials, including news articles, video lectures, professional knowledge courses, and cultural education resources. In addition, it incorporates multiple gamification features such as point systems, badges, ranking lists, and daily learning tasks, which are designed to motivate users to engage in continuous learning. In addition, the platform places strong emphasis on content quality, requiring many learning resources to be produced or reviewed by domain experts. These characteristics make LPP a representative case for examining the interaction between content quality, gamification design, and learning performance in online learning environments.

Participants were recruited using convenience sampling, a widely adopted non-probability sampling approach in survey-based research when access to a complete sampling frame is limited (Hair et al., 2019). This approach is particularly suitable for studies involving online platform users, where the population of interest is difficult to fully identify and access (Etikan et al., 2016). To ensure the quality and accuracy of the questionnaire, measurement items were adapted from established scales reported in prior studies and underwent a rigorous translation–back-translation procedure to maintain semantic equivalence. The questionnaire was reviewed by two professors to ensure clarity and content validity before distribution. It was then distributed to experienced users of the Learning Progress Platform (LPP) who had sufficient familiarity with the platform’s learning functions and gamification features. To encourage participation, respondents were provided with incentives and periodic reminders during a two-week data collection period. A strict data screening procedure was conducted to eliminate incomplete or invalid responses. As a result, 207 valid questionnaires were obtained from 400 distributed surveys, yielding an effective response rate of 51.75%. These procedures ensured the credibility and reliability of the data used for subsequent analysis.

### 3.2 Measurement of variables

Research data were collected using a structured questionnaire to test the proposed hypotheses. All constructs in the research model were measured using multi-item scales adapted from well-established studies, ensuring the content validity of the measurement instruments. Minor wording modifications were made to align the items with the context of online learning platforms. All items were measured on a five-point Likert scale, ranging from 1 (“strongly disagree”) to 5 (“strongly agree”).

Content quality was measured through three dimensions—authority, knowledge, and timeliness—based on established information system quality scales (DeLone and McLean, 1992; Wixom and Todd, 2005; Urbach and Ahlemann, 2010). Authority captures the credibility and reliability of the information sources on the platform. Knowledge reflects the depth and usefulness of the learning content, while timeliness evaluates whether the platform provides up-to-date and relevant information.

Gamification design was measured through two dimensions: fun and socialization. This decision is based on two key perspectives. First, from a theoretical standpoint, recent research emphasizes that enjoyment and social interaction are the most prominent and effective elements of gamification in educational contexts (Deterding et al., 2011; Murillo-Zamorano et al., 2023). While traditional game mechanics, such as points, badges, and leaderboards, primarily offer extrinsic rewards, increasing evidence suggests that it is the experiential aspects—specifically intrinsic enjoyment and social connectedness—that truly drive sustained learner engagement and motivation (Li et al., 2024a; Tseng et al., 2024; Murillo-Zamorano et al., 2023). Second, from a contextual perspective, this choice aligns closely with the specific design features of the target platform. The Learning Power Platform (LPP) is a public-service, lifelong learning environment. Unlike highly competitive commercial e-learning systems, LPP is purposefully designed to foster collaborative community interaction and promote continuous self-improvement, rather than intense, zero-sum competition among learners. Therefore, measuring gamification through the dimensions of ‘fun’ and ‘socialization’ is not an oversimplification but a valid and contextually appropriate approach to understanding how gamification functions to engage users within this particular digital learning ecosystem.

External supervision was measured using items adapted from DeAngelo (1981), which assess the degree of monitoring, evaluation, and oversight provided by external institutions or regulatory bodies toward the platform. Learning performance was measured using a scale adapted from Wiggins (2006), capturing improvements in knowledge acquisition, critical thinking ability, and professional learning outcomes. Each construct was measured using at least three items, meeting the recommended minimum requirement for latent variable measurement in behavioral research. The detailed measurement items and their corresponding sources are presented in Appendix A.

## 4 Results

### 4.1 Descriptive statistical analysis and correlation analysis

We collected data on each user’s gender, age, level of education, profession, occupation, and completion of daily study tasks as control variables for the model presented in Table 1. The proportion of males in the sample is roughly 28.5%, and the proportion of females is approximately 71.5%, indicating a significant predominance of female participants. Regarding age distribution, the majority of users are under 20 years old, with a fairly balanced representation between those aged 20 to 40 and those over 40. This suggests a concentration of younger individuals in the tested population. In terms of educational background, over 91.9% of the participants hold a bachelor’s degree or higher, with 78.3% having a bachelor’s degree, 16.4% holding a master’s degree, and 5.3% having a college degree. This high level of education enhances the reliability of the survey results. The occupations of the participants span a wide range of industries, including students, office workers, and teachers. This diversity ensures the universality and broad applicability of the study. Additionally, 79.2% of the participants reported completing their daily study tasks, while 20.8% did not. This information provides insight into the engagement levels of the participants with their study tasks.

**Table 1:** Demographic and personal characteristics of participants.

Sample characterization	Frequency (*n* = 207)	(%)
Gender		
Male	59	28.5
Female	148	71.5
Age		
<20	113	55.4
20–40	74	35.7
>40	20	9.66
Education background		
College degree	11	5.3
Bachelor degree	162	78.3
Master’s degree	34	16.4
Major		
Science and engineering	141	68.1
Literature and history	66	31.9
Occupation		
Office worker	115	55.6
Teacher	31	15.0
Student	61	29.5
Task completion		
Yes	164	79.2
No	43	20.8

In our study, Pearson correlation analysis was utilized to investigate the extent of correlation among the variables, and the results are presented in Table 2. As evidenced by the results of the analysis presented in Table 2, the standardized correlation coefficients between each dimension are less than the square root of the AVE value corresponding to the dimension. This observation supports the conclusion that the dimensions exhibit satisfactory discriminant validity (Fornell and Larcker, 1981; Kim et al., 2012). This suggests that the overall scale falls within an acceptable range. Nevertheless, relying solely on the results of the correlation analysis provides a preliminary indication of the relationships between the variables. Further validation of the specific path of influence and hypotheses is required through regression analysis.

**Table 2:** Means, SD, and correlations (*n* = 207).

Construct	Mean	SD	1	2	3	4	5	6	7
AUT	4.45	0.53	0.887						
KNO	4.08	0.71	0.124	0.863					
SOC	3.60	0.90	0.083	0.106	0.849				
TIM	3.61	0.84	−0.059	0.209^**^	0.244^**^	0.791			
FUN	4.18	0.70	0.356^**^	0.149^*^	0.166^*^	0.510^**^	0.832		
EXT	3.82	0.72	−0.096	0.025	0.324^**^	0.390^**^	0.222^**^	0.812	
LP	4.28	0.57	0.391^**^	0.169^*^	−0.002	0.120	0.157^*^	0.006	0.784

^**^p < 0.05; ^*^p < 0.10. AUT, KNO, SOC, TIM, FUN, EXT, and LP are the abbreviations of authority, knowledge, timeliness, fun, socialization, external supervision, and learning performance; the square roots of AVEs are in the diagonal.

### 4.2 Common method Bias

To address potential common method variance (CMV), we applied statistical techniques as recommended in prior research (Li et al., 2020a). Specifically, Harman’s single-factor test was conducted by loading all items into an exploratory factor analysis without rotation. The results showed that the largest factor accounted for only 27.53% of the variance, well below the 50% threshold, suggesting that CMV is unlikely to pose a significant concern (Podsakoff et al., 2003).

### 4.3 Validity and reliability

To assess the reliability and validity of the measurement instruments, several statistical tests were conducted. First, Cronbach’s alpha coefficients were calculated to evaluate internal consistency reliability. All constructs exceeded the recommended threshold of 0.70, indicating satisfactory reliability. Second, composite reliability (CR) and average variance extracted (AVE) were calculated to assess convergent validity (Li et al., 2019). The CR values were above 0.70 and the AVE values exceeded the recommended threshold of 0.50, confirming adequate convergent validity (Table 3; Li et al., 2020b). These results provide evidence of convergent validity of the measurement model (Fornell and Larcker, 1981).

**Table 3:** Results of reliability analysis of research variables.

Factor	Loadings	AVE	CR	Cronbach’s alpha
Content quality				
Authority (AUT)		0.787	0.917	0.863
AUT1	0.864			
AUT2	0.78			
AUT3	0.829			
Timeliness (TIM)		0.744	0.897	0.82
TIM1	0.762			
TIM2	0.772			
TIM3	0.818			
Knowledgeable (KNO)		0.72	0.885	0.804
KNO1	0.928			
KNO2	0.645			
KNO3	0.725			
Gamification design				
Socialization (SOC)		0.626	0.833	0.7
SOC1	0.713			
SOC2	0.757			
SOC3	0.79			
FUN		0.692	0.871	0.774
FUN1	0.795			
FUN2	0.756			
FUN3	0.654			
External supervision (EXT)		0.66	0.853	0.732
EXT1	0.774			
EXT2	0.588			
EXT3	0.735			
Learning performance (LP)		0.614	0.864	0.788
LP1	0.674			
LP2	0.618			
LP3	0.812			
LP4	0.679			

AVE, average variance extracted; CR, composite reliability index.

To assess the construct validity of the measurement model, confirmatory factor analysis (CFA) was conducted using AMOS. CFA is widely used to evaluate whether the observed measurement items adequately represent the underlying theoretical constructs (Hair et al., 2019). The results indicated that the measurement model demonstrated an acceptable level of fit with the data. Specifically, the model fit indices were satisfactory (*χ*^2^ = 282.641, df = 188, *χ*^2^/df = 1.503, NFI = 0.86, IFI = 0.948, TLI = 0.928, CFI = 0.946, RMSEA = 0.049). According to commonly accepted guidelines, a *χ*^2^/df ratio below 3, CFI, TLI, and IFI values above 0.90, and RMSEA values below 0.08 indicate a good model fit (Hu and Bentler, 1999; Hair et al., 2019). Therefore, the overall measurement model exhibited satisfactory goodness-of-fit. In addition, all standardized factor loadings ranged from 0.712 to 0.909, exceeding the recommended threshold of 0.50, and were statistically significant at the 0.01 level, indicating strong relationships between the observed variables and their corresponding latent constructs. These results provide evidence of convergent validity of the measurement model (Fornell and Larcker, 1981).

### 4.4 Hypothesis testing

To test the proposed hypotheses, hierarchical regression analysis was employed. This analytical approach is widely used in behavioral and management research to examine the relationships among variables and to test moderating effects (Hair et al., 2019). Compared with structural equation modeling, hierarchical regression allows researchers to directly assess the incremental explanatory power of interaction terms by entering variables into the regression model step by step. Specifically, control variables were entered in the first step, followed by the main independent variables. Interaction terms were then introduced in subsequent steps to examine moderating effects. To reduce potential multicollinearity, all continuous variables were mean-centered prior to creating interaction terms. This analytical strategy enabled us to examine the main effects, nonlinear relationships, and moderating effects proposed in the research model.

Specifically, all independent variables were standardized, and squared terms were calculated by multiplying the standardized variables by their respective values. Additionally, the squared term of the platform gamification design variable was introduced into the model to examine potential curvilinear effects. Multicollinearity diagnostics confirmed that the variance inflation factor (VIF) for all models was well below the threshold of 10, indicating that multicollinearity was not a concern. Subsequently, stepwise hierarchical regression was employed to test the research hypotheses. The results are summarized in Table 4. From Model 1 to Model 4, the *R*^2^ values gradually increased (from 0.011 to 0.313), indicating that the explanatory power of the model strengthened with the addition of variables. The adjusted *R*^2^ values were consistent with the trend of *R*^2^ changes. Additionally, the *F*-values for all models were significant, indicating that the overall regression was statistically significant. Stepwise hierarchical regression was then used to test the research hypotheses.

**Table 4:** Results of regression analysis.

Variable	Learning performance			
	Model 1	Model 2	Model 3	Model 4
Explanatory variable				
GAM		0.221^**^	0.188^*^	0.336^***^
GAM^2^			−0.118^↑^	−0.167^**^
INF		0.451^***^	0.451^***^	0.496^***^
GAM^2^ * EXT				−0.287^**^
INF * EXT				−0.062
Constant	−0.014	0.393^**^	0.395^**^	0.346^**^
Control variable				
Sex				
Female	0.098	−0.082	−0.055	−0.062
Age				
20–40	0.05	−0.18	−0.134	−0.046
>40	0.079	−0.004	0.004	0.012
Education				
Bachelor	−0.004	−0.108	−0.096	−0.081
*R* ^2^	0.011	0.267	0.279	0.313
Adjusted *R*^2^	−0.009	0.245	0.255	0.282
*F*-value	0.549	12.072^***^	10.945^***^	9.941^***^

^↑^*p* < 0.1, ^***^p < 0.01; ^**^p < 0.05; ^*^p < 0.10.

Model 1 served as the baseline, examining the impact of control variables (e.g., gender, age, and education) on learning performance. The results indicated no significant influence of these variables, providing a neutral foundation for subsequent analyses. Model 2 introduced the content quality and platform gamification design variables. As anticipated, the regression results showed a significant positive relationship between content quality and learning performance (*β* = 0.451, *p* < 0.001), confirming that higher content quality directly enhances learning performance. Additionally, a significant positive relationship was revealed between gamification and learning performance (*β* = 0.221, *p* < 0.05). Hypothesis 1 was supported. The model’s *F*-value met the significance threshold (*p* < 0.001), and the coefficient of determination *R*^2^ indicated improved explanatory power.

Model 3 expanded upon these findings by incorporating the squared term of gamification design. Results demonstrated a significant negative effect of the squared term (*β* = −0.118, *p* < 0.10), confirming an inverted U-shaped relationship between gamification and learning performance. This indicates that while moderate levels of gamification enhance learning outcomes, excessive gamification may diminish its positive effects. Additionally, since the axis of symmetry of the curve was located within the range of observed gamification values, this curvilinear relationship was validated, supporting hypothesis H2. Based on the inverted U-shaped relationship validation, this study calculated the peak point of the inverted U-shaped relationship between gamification design and learning performance using partial regression coefficients. For standardized variables, the inverted U-shaped relationship formula is: Y = a + bX + cX^2^ (where Y represents learning performance, X represents gamification design, b is the regression coefficient of gamification design, and c is the regression coefficient of the squared term of gamification design). The formula for calculating the peak point is: X = −b/(2c). According to the regression analysis results (Model 3), b = 0.221, c = −0.118. Substituting these values into the formula yields the peak point X = −0.221/(2 × −0.118) ≈ 0.932 (standardized value). This standardized value corresponds to an upper-middle level of gamification design. Specifically, when the platform’s gamification design reaches an upper-middle level characterized by “moderate elements of fun and social interaction, without excessive competition or complex reward rules,” its positive impact on learning performance is maximized. When the gamification design level exceeds this peak point, indicating overgamification, the effect on learning performance shifts from positive to negative.

While moderate gamification enhances motivation, excessive game mechanics impose extraneous cognitive load, distracting learners from the core educational content. Based on our regression model Y = 0.221X − 0.118X^2^, the optimal peak for learning performance occurs when gamification intensity is approximately 0.93 (standardized value), beyond which the benefits begin to diminish.

**Figure 2:**
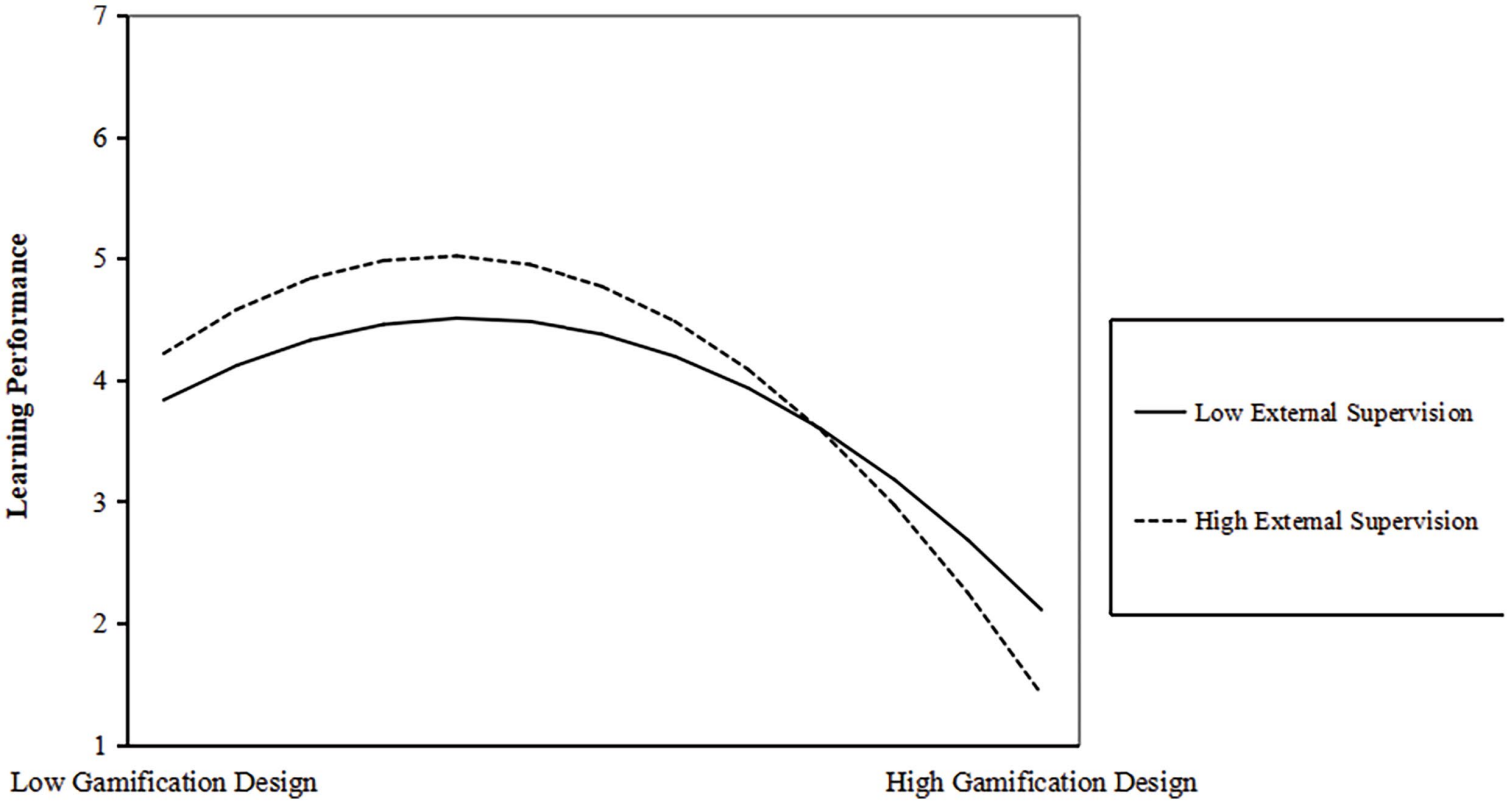
Inverted U-shape and moderating effect.

For ease of visualization, we illustrated this relationship in Figure 2. As shown, as the level of platform gamification design increases, users’ learning performance initially improves and then decreases, presenting an inverted U-shaped trend. We believe hypothesis H2 is supported.

Finally, the moderating effects of external supervision were tested in Models 3 and 4. In Model 3, the interaction between the squared term of gamification and external supervision was significant (*β* = −0.287, *p* < 0.001). This finding suggests that external supervision moderates the inverted U-shaped relationship between gamification and learning performance, mitigating the negative effects of over-gamification. This result validates hypothesis H4. Conversely, in Model 4, the interaction term between content quality and external supervision was not significant, indicating that external supervision does not moderate the relationship between content quality and learning performance. Thus, hypothesis H3 is not supported. Additionally, the visual results for the interaction between the squared term of gamification and external supervision are shown in Figure 2. External supervision shifts the originally inverted U-shaped curve upward, enhancing learning performance. However, surprisingly, as the level of gamification increases, external supervision exacerbates the negative effects on learning performance.

Our results clarify that the role of external supervision is contingent on the stage of gamification. In the ascending phase, supervision functions as a supportive guide that reinforces the motivational effects of rewards. However, in the descending phase, high levels of supervision exacerbate the negative consequences of over-gamification. More specifically, this study further examines the moderating effect of external supervision across the ascending and descending stages of the inverted U-shaped relationship between gamification design and learning performance. First, in the ascending phase (X < 0.932), the interaction coefficient between external supervision and gamification design is 0.189 (*p* < 0.05). This result indicates that external supervision amplifies the positive effect of moderate gamification on learning performance by setting learning goals and guiding the appropriate use of gamified elements. Under such conditions, learners are more likely to focus on learning tasks while being motivated by gamification incentives. Second, in the descending phase (X > 0.932), the interaction coefficient between external supervision and the squared term of gamification design is −0.287 (*p* < 0.001). This finding suggests that although external supervision strengthens the evaluation of learning outcomes and attempts to correct learning behaviors driven by gamified incentives, excessive gamification has already caused cognitive overload among learners. As a result, the additional goal pressure introduced by external supervision further increases cognitive burden, thereby intensifying the negative impact of over-gamification on learning performance.

## 5 Discussion

This study, based on Social Learning Theory, investigates the interactive effects of content quality, gamification design, and external supervision on learning performance in an online learning platform, revealing that content quality is paramount, gamification has an inverted U-shaped relationship with performance, and external supervision moderates the impact of gamification.

In recent years, online learning platforms have increasingly focused on integrating engaging gamification elements and delivering high-quality content to improve user experience and outcomes (Huang et al., 2021; Singh and Thurman, 2019). However, this dual emphasis often leans heavily toward gamification, sometimes at the expense of content quality (Kam and Umar, 2023; Al-Fraihat et al., 2020; Zhang et al., 2024; Lopez and Tucker, 2019). This imbalance raises critical questions about the relative contributions of content quality and gamification design to learning performance. Building upon prior research, our findings offer a more nuanced perspective by exploring the optimal balance between these two factors (Sailer and Sailer, 2021; Zainuddin et al., 2020). Furthermore, we contextualize these dynamics by examining broader environmental and social influences—particularly the regulatory role of external supervision—as conceptualized within SLT (Zuchowski, 2015).

Aligned with prior research, our results confirm that content quality is a key driver of learning performance on e-learning platforms (DeLone and McLean, 2003). By delving deeper into the dimensions of content quality—authority, timeliness, and knowledge—our study emphasizes that these attributes are essential for fostering user satisfaction and effective knowledge acquisition. Given that the primary objective for users on e-learning platforms is practical knowledge application, ensuring high standards of content quality remains paramount.

In exploring the inverted U-shaped relationship between gamification and learning performance, our findings illuminate the dual-edged nature of gamification in online education. Gamification elements initially enhance engagement and motivation, driving active participation and interest. However, as gamification features become overly prominent, their motivational effects wane, leading to a decline in attention and effort toward substantive learning. From the perspective of cognitive psychology, this inverted U-shaped relationship can be explained by Cognitive Load Theory. Individuals have limited cognitive processing capacity when performing learning tasks. Moderate gamification elements—such as points, badges, and social interaction features—can attract learners’ attention and enhance engagement (Chen et al., 2023; Seaborn and Fels, 2015), thereby directing cognitive resources toward the learning task and improving learning outcomes (Kuo and Chuang, 2016; Al-Fraihat et al., 2020). However, excessive gamification introduces numerous non-essential game elements, such as complex reward systems or frequent competitive feedback, which may occupy learners’ attention and cognitive resources (Pitthan and Witte, 2025). As a result, learners experience increased extraneous cognitive load, which can lead to cognitive overload and ultimately reduce learning performance (Berkling and Thomas, 2013; Riar et al., 2022).

Our investigation into the moderating role of external supervision reveals complex dynamics. While external supervision does not significantly moderate the relationship between content quality and learning performance, this may stem from the inherent strengths of high-quality content. Attributes such as authority, timeliness, and knowledge likely overshadow the influence of external supervision, making it less impactful in altering the direct effects of content quality (Yang et al., 2017; Li et al., 2024a). The findings reveal that the moderating effect of external supervision on gamification acts as a double-edged sword depending on the specific stage of the inverted U-shaped relationship. During the ascending stage of moderate gamification, supervision functions as a positive catalyst that guides learners to effectively utilize gamified incentives, thereby strengthening the enhancement of learning performance. Conversely, during the descending stage of excessive gamification, strict supervision becomes a detrimental stressor that intensifies cognitive overload and consequently amplifies the negative impact of over-gamification on learning outcomes (Krath et al., 2021). Grounded in SLT’s triadic reciprocal determinism, external supervision exerts different effects across the stages of the U-curve (Bandura, 1991). In the ascending stage, where gamification remains at a moderate level, external supervision functions as an effective environmental regulation mechanism (Abbey and Meloy, 2017). By setting clear learning goals and providing structured feedback, supervision helps learners focus their attention and ensures that the vicarious reinforcement provided by gamified incentives is directed toward educational tasks, thereby strengthening the positive impact of moderate gamification (An and Wulf, 2024; Roslan et al., 2023). However, in the descending stage—where gamification exceeds the optimal level—the dynamics shift according to Cognitive Load Theory (Sweller, 2020). Excessive gamification inherently introduces high extraneous cognitive load. When paired with strong external supervision, which also requires learners to allocate cognitive resources to process additional monitoring and performance expectations, the combined demands overload the learners’ limited working memory capacity. Rather than effectively regulating behavior, intense supervision compounds the cognitive burden and diminishes learner autonomy, thereby amplifying the negative impact of over-gamification on learning performance (Coelho et al., 2025). These findings suggest that the effectiveness of supervision is contingent on the level of gamification, highlighting the importance of dynamically adjusting supervisory intensity according to the stage of gamification design.

This study makes significant contributions to the literature on gamification and learning performance in online education by bridging the knowledge and interactive dimensions of e-learning platforms. First, unlike prior research that often emphasizes one factor over the other—either focusing on gamification to boost user engagement (Smith and Abrams, 2019) or prioritizing content quality to ensure substantive learning (Zhang and Huang, 2024; Ho et al., 2023)—our study underscores the equal importance of both. By examining the interplay between content quality and gamification design, this research provides a more holistic perspective on their combined influence on learning performance, advancing our understanding of how these elements synergistically shape educational outcomes. Second, this research provides nuanced, theory-based empirical insights into the boundary conditions of gamification design. Addressing the need for a cautious interpretation of non-linear effects, our findings suggest a threshold dynamic grounded in Cognitive Load Theory. The observed inverted U-shaped relationship indicates that while moderate gamification enhances user engagement and directs attention toward learning tasks, excessive reliance on game mechanics introduces extraneous cognitive load that competes with working memory. These results align with prior studies highlighting the potential risks of over-gamification (Ho et al., 2023; Kwon and Özpolat, 2021), while providing new insights into the threshold at which gamification begins to hinder learning performance. Third, this study extends the application of Social Learning Theory (SLT) in digital learning environments by elucidating the regulatory function of external supervision. While traditional SLT emphasizes learning through peer or instructor observation, our research deepens the explanation of institutional oversight as an integral part of SLT’s framework of triadic reciprocal determinism (Justin and Joy, 2024). We demonstrate that external supervision is not merely a passive environmental factor, but a crucial mechanism for moderating and re-aligning learners’ self-regulatory processes. By examining the interaction between external supervision, gamification design, and content quality, our findings highlight its dual role in both enhancing the benefits of gamification and mitigating its potential drawbacks. This underscores the importance of aligning gamification with core educational objectives, rather than treating it as a mere engagement tool. When gamification elements shift from being motivating to distracting, external supervision intervenes to recalibrate the learner’s cognitive focus, counteracting the disruptive influence of extraneous stimuli introduced by platform gamification. Ultimately, by integrating the internal cognitive limits from Cognitive Load Theory with the external institutional scaffolding of our extended SLT framework, this study constructs a cohesive theoretical model. It demonstrates that the efficacy of digital learning fundamentally relies on a delicate equilibrium among high-quality content, calibrated gamification, and robust environmental supervision. Specifically, high-quality content guarantees the substantive value of learning, calibrated gamification sustains intrinsic motivation without triggering cognitive overload, and external supervision provides the critical scaffolding to keep learners’ self-regulation on track.

The findings of this study offer valuable guidance for the development and optimization of online learning platforms. First, to avoid an imbalanced focus on either gamification or content quality, platform designers must strive for a synergistic approach that harmonizes these two critical dimensions. Second, developers should carefully design gamification elements to ensure that they enhance engagement without compromising educational outcomes. Gamification features should be calibrated to avoid overemphasizing entertainment at the expense of substantive learning. Third, platform administrators must prioritize content quality by rigorously evaluating its authority, timeliness, and knowledge value. Maintaining high standards of content quality is essential to meet users’ primary educational needs and sustain long-term engagement. Finally, robust mechanisms for assessing platform learning performance should be implemented to inform effective external supervision strategies. External monitoring should be evidence-based and tailored to address potential negative consequences of over-gamification. By providing structured guidance and oversight, external supervision can help users stay focused on their learning goals, mitigating distractions and maintaining motivation throughout the learning process.

## 6 Conclusion

This study investigates the influence of content quality and gamification design on learning performance in online learning platforms while exploring the moderating role of external supervision through the lens of SLT. The findings emphasize that content quality is a critical and consistent driver of learning performance, highlighting its centrality in online educational settings. In contrast, the relationship between gamification design and learning performance follows an inverted U-shaped curve, indicating that gamification can be effective within an optimal range, but excessive gamification leads to diminishing returns and even negative impacts on learning outcomes. Furthermore, external supervision is found to enhance the positive effects of gamification when it is within the optimal range; however, when gamification exceeds this threshold, external supervision exacerbates the negative consequences. Notably, external supervision does not significantly moderate the relationship between content quality and learning performance, reinforcing the notion that content quality remains the dominant factor in shaping learning outcomes.

This study has several limitations that should be acknowledged. First, while the use of cross-sectional, self-reported survey data effectively captures user behaviors within a specific timeframe, it inherently bounds our capacity to draw definitive causal inferences. Although our theoretical framework robustly supports the proposed directionality, future research incorporating longitudinal tracking or experimental designs would be valuable to further solidify these causal pathways. Second, the sample was drawn from a single digital platform, yielding a demographic profile predominantly composed of highly educated (91.9% holding a bachelor’s degree or higher) and female (71.5%) participants. While this composition accurately reflects the active user base engaged in self-directed learning on platforms like LPP, it introduces certain contextual boundaries. For instance, prior literature indicates that female learners may exhibit greater responsiveness to social gamification, whereas male learners might be more attuned to competitive elements (Lopez and Tucker, 2019). Consequently, future studies could build upon this foundational model by examining these dynamics across more heterogeneous demographic profiles to continuously expand its generalizability. Finally, the operationalization of gamification was purposefully focused on the dimensions of enjoyment and socialization, aligning closely with the target platform’s non-competitive design ethos. As such, the observed inverted U-shaped relationship is most accurately interpreted within the context of social and entertainment-oriented gamification. It is plausible that learning environments heavily reliant on aggressive competition or penalty-based mechanics might exhibit different threshold dynamics, which presents an intriguing avenue for future comparative research.

## Data Availability

The original contributions presented in the study are included in the article/Supplementary material, further inquiries can be directed to the corresponding author.
