# Supplementary material for: Can gamification really enhance learning performance? The importance of balancing gamification design and content quality
Source: Front Sociol. 2026 Jun 19;11:1740629. doi: 10.3389/fsoc.2026.1740629 (PMC13328485; doi:10.3389/fsoc.2026.1740629)
Supplement: Supplementary file 1 [file Supplementary_file_1.DOCX]

Supplementary Material

# Supplementary Figures and Tables

For more information on Supplementary Material and for details on the different file types accepted, please see [here](https://www.frontiersin.org/guidelines/author-guidelines#supplementary-material).

## Supplementary Figures

**Supplementary Figure 1.** Research Model.

**
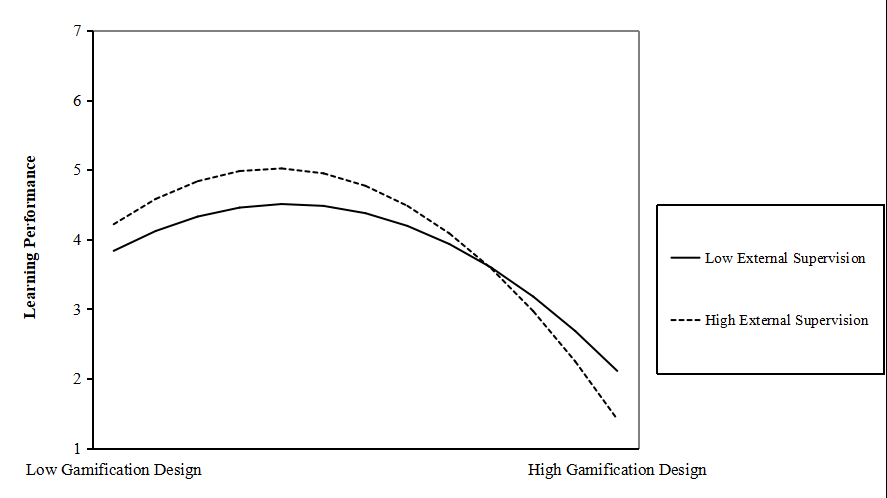
**

**Supplementary Figure 2.** Inverted U-shape and Moderating Effect

- 1. **Supplementary Tables**

**Table 1.** Demographic and Personal Characteristics of Participants.

| **Sample Characterization** | **Frequency (n = 207)** | **(%)** |
| --- | --- | --- |
| Gender |  |  |
| Male | 59 | 28.5 |
| Female | 148 | 71.5 |
| Age |  |  |
| <20 | 113 | 55.4 |
| 20-40 | 74 | 35.7 |
| >40 | 20 | 9.66 |
| Education background |  |  |
| College Degree | 11 | 5.3 |
| Bachelor Degree | 162 | 78.3 |
| Master's Degree | 34 | 16.4 |
| Major |  |  |
| Science and Engineering | 141 | 68.1 |
| Literature and History | 66 | 31.9 |
| Occupation |  |  |
| Office Worker | 115 | 55.6 |
| Teacher | 31 | 15.0 |
| Student | 61 | 29.5 |
| Task Completion |  |  |
| Yes | 164 | 79.2 |
| No | 43 | 20.8 |

**Table 2.** Means, SD, and Correlations (N=207).

|  | **Mean** | **SD** | **1** | **2** | **3** | **4** | **5** | **6** | **7** |
| --- | --- | --- | --- | --- | --- | --- | --- | --- | --- |
| AUT | 4.45 | 0.53 | 0.887 |  |  |  |  |  |  |
| KNO | 4.08 | 0.71 | 0.124 | 0.863 |  |  |  |  |  |
| SOC | 3.60 | 0.90 | 0.083 | 0.106 | 0.849 |  |  |  |  |
| TIM | 3.61 | 0.84 | -0.059 | 0.209** | 0.244** | 0.791 |  |  |  |
| FUN | 4.18 | 0.70 | 0.356** | 0.149* | 0.166* | 0.510** | 0.832 |  |  |
| EXT | 3.82 | 0.72 | -0.096 | 0.025 | 0.324** | 0.390** | 0.222** | 0.812 |  |
| LP | 4.28 | 0.57 | 0.391** | 0.169* | -0.002 | 0.120 | 0.157* | 0.006 | 0.784 |

**p < 0.05; *p < 0.10. AUT, KNO, SOC, TIM, FUN, EXT, and LP are the abbreviations of authority, knowledge, timeliness, fun, socialization, external supervision, and learning performance; the square roots of AVEs are in the diagonal.

**Table 3.** Results of Reliability Analysis of Research Variables.

| **Factor** | **Loadings** | **AVE** | **CR** | **Cronbach's alpha** |
| --- | --- | --- | --- | --- |
| **Content Quality** |  |  |  |  |
| Authority (AUT) |  | 0.787 | 0.917 | 0.863 |
| AUT1 | 0.864 |  |  |  |
| AUT2 | 0.78 |  |  |  |
| AUT3 | 0.829 |  |  |  |
| Timeliness ( TIM ) |  | 0.744 | 0.897 | 0.82 |
| TIM1 | 0.762 |  |  |  |
| TIM2 | 0.772 |  |  |  |
| TIM3 | 0.818 |  |  |  |
| Knowledgeable (KNO) |  | 0.72 | 0.885 | 0.804 |
| KNO1 | 0.928 |  |  |  |
| KNO2 | 0.645 |  |  |  |
| KNO3 | 0.725 |  |  |  |
| **Gamification Design** |  |  |  |  |
| Socialization (SOC) |  | 0.626 | 0.833 | 0.7 |
| SOC1 | 0.713 |  |  |  |
| SOC2 | 0.757 |  |  |  |
| SOC3 | 0.79 |  |  |  |
| FUN |  | 0.692 | 0.871 | 0.774 |
| FUN1 | 0.795 |  |  |  |
| FUN2 | 0.756 |  |  |  |
| FUN3 | 0.654 |  |  |  |
| External Supervision ( EXT ) |  | 0.66 | 0.853 | 0.732 |
| EXT1 | 0.774 |  |  |  |
| EXT2 | 0.588 |  |  |  |
| EXT3 | 0.735 |  |  |  |
| Learning Performance (LP) |  | 0.614 | 0.864 | 0.788 |
| LP1 | 0.674 |  |  |  |
| LP2 | 0.618 |  |  |  |
| LP3 | 0.812 |  |  |  |
| LP4 | 0.679 |  |  |  |

* AVE = average variance extracted; CR = composite reliability index.

**Table 4.** Results of Regression Analysis

|  | Learning Performance | | | |
| --- | --- | --- | --- | --- |
|  | Model 1 | Model 2 | Model 3 | Model 4 |
| Explanatory variable |  |  |  |  |
| GAM |  | 0.221** | 0.188* | 0.336*** |
| GAM^2 |  |  | -0.118↑ | -0.167** |
| INF |  | 0.451*** | 0.451*** | 0.496*** |
| GAM^2*EXT |  |  |  | -0.287** |
| INF*EXT |  |  |  | -0.062 |
| Constant | -0.014 | 0.393** | 0.395** | 0.346** |
| Control variable |  |  |  |  |
| Sex |  |  |  |  |
| Female | 0.098 | -0.082 | -0.055 | -0.062 |
| Age |  |  |  |  |
| 20-40 | 0.05 | -0.18 | -0.134 | -0.046 |
| >40 | 0.079 | -0.004 | 0.004 | 0.012 |
| Education |  |  |  |  |
| Bachelor | -0.004 | -0.108 | -0.096 | -0.081 |
| R2 | 0.011 | 0.267 | 0.279 | 0.313 |
| Adjusted R2 | -0.009 | 0.245 | 0.255 | 0.282 |
| F-value | 0.549 | 12.072*** | 10.945*** | 9.941*** |
| ↑p < 0.1, ***p < 0.01; **p < 0.05; *p < 0.10. | | | | |

**Appendix A**

**Table A1** Measurement items

| **Content Quality** | Authority  Urbach et al. (2010) | The information on the learning platform is released by authoritative official organizations. |
| --- | --- | --- |
|  |  | The sources of information on the learning platform are strictly controlled. |
|  |  | The sources of information on the learning platform are subject to rigorous oversight. |
|  | Knowledge  Delone & Mclean (1992) | The content provided by the platform is highly valuable. |
|  |  | The platform content reflects a deep understanding of the subject matter. |
|  |  | The content aligns with mainstream values and norms. |
|  | Timeliness  Wixom and Todd (2005) | I can use the platform to search for the knowledge that I am interested in; |
|  |  | I can use the platform to understand the current hot events. |
|  |  | The platform can timely provide what I want to browse. |
| **Gamification Design** | Fun  Murillo-Zamorano et al. (2023) | The platform's gamified design (e.g., badges or progress tracking) enhances my overall learning experience. |
|  |  | Gamified elements make searching for knowledge fun and engaging. |
|  |  | The platform offers timely, personalized recommendations with fun rewards. |
|  | Socialization  Deterding et al.(2011) | The platform's gamified social features like group challenges or team rankings make learning more interactive and fun. |
|  |  | Competing with or collaborating with others through gamified activities enhances my learning experience. |
|  |  | The platform's social rewards like badges or recognition motivate me to engage with peers. |
| **External Supervision** DeAngelo et al.(1981) | | External supervision maintains independent oversight of the online learning platform. |
|  |  | External supervision regularly reviews affiliated online learning platforms. |
|  |  | External supervision promptly identifies issues and promotes improvements. |
| **Learning Performance** Wiggins (2006) | | My studies on this platform have significantly expanded my professional knowledge. |
|  |  | The platform's content recommendations have enhanced my understanding of diverse social cultures. |
|  |  | This learning experience has improved my critical thinking skills in professional contexts. |
|  |  | Studying on this platform has deepened my knowledge across various areas, including the world and nature. |
